# Basic and translational endocrinology

**DOI:** 10.20945/2359-4292-2024-0401

**Published:** 2024-11-06

**Authors:** Maria Tereza Nunes

**Affiliations:** 1 Universidade de São Paulo São Paulo SP Brasil Universidade de São Paulo, Fisiologia e Biofísica, São Paulo, SP, Brasil

This special issue of the *Archives of Endocrinology and Metabolism* is dedicated to Basic and Translational Research. It presents diverse articles and reviews with different focuses, which show the broad coverage of subjects that permeate this topic.

Some of them, including a minireview (1), are related to endocrine disruptors compounds (EDCs), that are known to impact all forms of life that live in the planet and the environment, therefore affecting the reproduction, sustainability, and development (2). Part of the EDCs, as reported herein, induce epigenetic transgenerational effect that alter disease susceptibility later in life (3). These studies, altogether, strengthen the importance of the strict regulation of the EDCs to ensure protection of human health and the environment (4).

One manuscript addresses strategies for investigating the biology of thyroid cancer, whose incidence rates have continuously increased. Therefore approaches for a better understanding and elucidation of the molecular mechanisms that underlie cancer development are highly desirable and of paramount importance as well as challenging. This paper presents methodologies based on genetic engineering, like the CRISPR/Cas9 system, for gene inactivation and genome editing that can disrupt target gene by generating insertions and deletions (5). It is important to highlight that this methodology can be applied to many research topics pertinent to endocrinology, opening opportunities for gene therapy for the study of other diseases.

Another study shows that triiodothyronine induces the expression of amphiregulin (AREG) in cancer breast cells by a noncanonical mechanism involving interaction of the hormone to binding sites at the plasma membrane (6). This is a very interesting finding since this protein is known to increase cancer progression, and it has been suggested as a promised prognostic marker of metastases, giving support to other literature data that link T3 with breast cancer aggressiveness.

Considering that diabetes mellitus is one of the most common endocrinopathies, strategies to ameliorate this condition are always a focus of attention. This issue presents two papers that address this topic. One of them explores the effect of the acute aerobic exercise on circulating endothelial progenitor cells and inflammatory markers in type 1 diabetes (7) and the other shows that metformin, a widely used antidiabetic medication, improves neuropathy in diabetic rats by reducing autophagy through an AMPK-dependent manner (8).

A study of sexual differences on reactive oxygen species (ROS) expression and antioxidant response of the adipose tissue of rats is also present in this issue (9). This is a relevant topic taking into account that imbalance between the production of ROS and the body's antioxidant capacity leads to oxidative stress, which is linked to several chronic diseases, as diabetes and atherosclerosis, as well as to DNA damage, which is associated with cancer development. Therefore, strategies to prevent oxidative stress are very useful to avoid lifestyle-related diseases and for a better quality of life.

Another interesting study showing that the aromatase gene variant CYP19A1 rs10046 is associated with cardiovascular risk in postmenopausal women highlights the importance of the molecular biology strategies to enable genomic research, and to establish correlations like this, which is important for prediction and prevention of cardiovascular complications in this period of life (10). In the same context, a study presented herein reinforces the anti-inflammatory effects of estrogen and its role in modulating baroreflex function in ovariectomized rats, which in addition to its vasodilatory properties lead us to better understand why postmenopausal women have increased risk of coronary heart disease (11).

Human gender identity is a very complex and fascinating theme, which has received most attention in recent times considering the great repercussion it causes in mental health and in the society. Hence, a review about its genetic and hormonal basis could not be absent from the present edition (12).

Another significant topic that is always present in the biomedical literature is the control of body weight, considering the increasing global epidemic of overweight and obesity and their metabolic outcomes, as type 2 diabetes, cardiovascular diseases and cancer. A review of this essential topic focusing on its hypothalamic control, including old and new partners, associated with perspectives to treat obesity is also present in this special issue, and will provide a better understanding of its present status (13).

An additional paper was developed in Prop1df/df dwarf mice, in which the pituitary growth and cell specification are impaired. This study showed that their treatment with GH and levothyroxine restores the fertility as shown by the studies of the peripheral reproductive parameters (14).

A case report was also included in this issue, in which molecular markers were used in association to ultrasonography to unveil a rare case of a lactotroph tumor (15).

Finally, an invited commentary on basic, translational, and clinical research was included in this special issue, authored by Dr. Peter Kopp, UNI, Division of Endocrinology, Diabetology and Metabolism, who described with great lucidity the importance of the cooperation among basic, translational and clinical research for advances in clinical practice (16).

All of manuscripts presented herein show the importance of using biochemical and molecular strategies in association with clinical and laboratory parameters for diagnostic purposes, therapy or to improve understanding of causal mechanisms that are behind the functioning of the human body in health and diseases.

Most of these advancements came from the progress of omics research (genomics, transcriptomics, proteomics, or metabolomics) generated by the molecular biology investigation, which led to the genome sequencing and provided novel approaches that have been widely used for different purposes by scientists and physicians (17).

Many other approaches are in use, with increased perspectives of enhancement of our knowledge in the field of regenerative medicine, since the demonstration that adult and specialized mouse cells could be reprogrammed and behave like embryonic stem cells (the induced pluripotent stem cells – ipsc cells), which could be converted into another cell type by the overexpression of lineage-specific factors (18).

In this context, the recent advances in the organoid technology have offered the possibility for creating full three-dimensional (3D) models that mimic the cellular heterogeneity, structure, and functions of the primary tissues, providing potential application in regenerative medicine, drug discovery, and precision medicine (19).

Concluding, I personally expect that in addition to all the information that the topics addressed herein provide, this special edition could help to change the misconception that the basic sciences research takes many years or even decades for the knowledge that is generated can have an application to clinical medicine. In fact, it can potentiate more rapid solutions to health challenges.

An additional challenge that should be overcome is to bridge the gap that exist between basic sciences faculty and clinical faculty. The improvement of their communication and integration will allow that basic sciences researchers face the difficulties related to the management of diseases, which will lead them to design and carry out appropriate experiments, resulting in discoveries that could be rapidly translated with potential uses and benefits for clinical practice and society.
